# Privacy of electronic health records: a review of the literature

**DOI:** 10.29173/jchla29496

**Published:** 2021-04-02

**Authors:** Katherine Gariépy-Saper, Nicholas Decarie

**Affiliations:** Graduate from McGill University

## Abstract

Privacy in the context of electronic health records (EHR) is an incredibly complex and multi-faceted topic within the LIS field. We conducted a narrative literature review and selected twenty-five articles published over the past fifteen years, which explore this topic from the perspectives of patients, doctors, medical librarians, informatics experts, records managers, and archivists. We identified themes that appeared consistently across the literature, as well as issues that differed across healthcare systems with varying levels of IT infrastructure. Significant changes have also taken place over time, especially with the development of technologies meant to protect privacy and make the widespread use of EHR possible. However, despite technological advances, many of the same problems of privacy ethics remain. Diverging opinions exist in the literature regarding how, and if, EHR systems should be established in light of these unresolved issues.

## Introduction

Health records have moved from the basement storage room under lock and key of major hospitals to digital clouds and hard-drives to increase accessibility and utility. Electronic health records^1^ (EHR) have the potential to transform communication within healthcare on multiple levels. This has been predicted since the 1960s and 70s when university medical centres began developing EHR systems that were a hybrid of digital and paper records systems [1]. As computer technology became more sophisticated and widespread in the 1990s and early 2000s, the literature surrounding EHR increased in volume and variety [1]. In the United States specifically, EHR featured in the 2004 State of the Union Address, as well as in a 2009 bill which mandated their use in hospitals that treat patients on government insurance [1].

One of the most profound impacts of EHR is the delocalization of health records, and quick delivery of information to any medical site [2]. Other changes include greater involvement of patients in their care (which is dependent on the rights and freedoms of the patient in their relevant jurisdiction) as well as creating the possibility of mass data collection for population research. However, EHR has numerous implications for privacy. The purpose of this paper is to review the literature surrounding EHR and privacy within the Library and Information Studies (LIS) field, and analyze how this issue has been studied. This paper will also identify major themes across the literature, and discuss areas that merit further consideration and research.

## Methods

### Search strategy

We performed a narrative review with the aim of having a broad array of sources from a variety of viewpoints, and from different countries. We selected the articles for our literature review through searching the databases: Library and Information Science Abstracts (LISA), and Library, Information Science and Technology Abstracts (LISTA). We chose these two because they are the most popular databases for Library and Information Science literature.

Similar to other traditional literature reviews, we did not use an exhaustive list of conceivable terminology or databases for the literature search. Instead, we opted to use the most relevant search terms to provide a general review of the subject. The Boolean OR functions allowed for the broadest possible recall of the two main concepts, which we hoped would provide a higher recall of results for our overall search. We combined the first concept, (privacy OR security OR confidentiality), with the second concept, (electronic medical records OR electronic health records OR EMR OR HER), using the Boolean AND function, so that the overall search would specifically retrieve literature containing both core subjects.

We restricted the results to the last fifteen years, from 2005 to 2020. We chose this date range because we wanted to represent and review recent literature, but we also wanted sufficient time depth to be able to analyze how the issues have developed over the years, as well as observe trends and changes. The starting year of 2005 was selected because EHR began to receive national attention and feature in federal policy in the United States, where the majority of selected papers originate. The authors agreed that limiting results to the last five or ten years was overly restrictive, and would ignore the time depth of the issues, and how they have evolved with technology.

### Selection

We selected a total of twenty-five articles to review, which came from a variety of countries, including Canada, the United States, the United Kingdom, Germany, Sweden, India, Iran, Zimbabwe, Uganda, Australia, China, and Singapore. We decided to include literature from around the world, because we consider health information to be a universal need, and recognize that research on the topic of privacy and EHR has been conducted around the world. Because of the universal aspects of health information and privacy, it was appropriate to include studies from multiple countries, in order to identify commonalities and differences, and to present a more complete review of the literature on this topic.

Although a certain degree of selection bias is inherent in the process of choosing literature for a review, we aimed to provide a fair representation of how EHR and privacy have been researched in the LIS field, and the ways this has changed over time. We selected an approximately equal number of articles from LISA and LISTA, to avoid any biases that could arise from LISTA's stronger technology focus. We included papers about specific technologies, security analyses for existing EHR systems, articles focused on ethics, qualitative studies of patient and healthcare provider communications, as well as archival and records management papers. Although the papers we selected represent diverse perspectives and areas of research within LIS, they all share electronic health records and privacy as their core themes. We excluded articles that were outside of our date range, and that only focused on one or the other theme (only privacy or only EHR), because these were outside of our review’s purview.

## Results

**Table 1:** Theme distribution across the literature.

	EHR Technology Focus	Anticipated Benefits	Patient-Doctor Trust	Privacy Ethics & Laws	EHR Ownership Question	Country-specific Focus	Publication Type
Gunter & Terry, 2005	No	Yes	No	Yes	No	United States; Australia	Viewpoint
McClanahan, 2008	Yes	Yes	No	Yes	Yes	United States	Viewpoint
Jones et al., 2010	Yes	Yes	No	Yes	Yes	United States	Task Force Paper
Baskaran et al., 2013	No	Yes	Yes	Yes	Yes	United Kingdom	Research Paper
Rodrigues et al., 2013	Yes	Yes	No	Yes	Yes	Spain; United States	Research Paper
Vodicka et al., 2013	No	Yes	Yes	Yes	No	United States	Research Paper
Campos-Castillo & Anthony, 2014	No	Yes	Yes	Yes	No	United States	Research Paper
Patel et al., 2015	No	Yes	Yes	Yes	No	United States	Research Paper
Vimalachandran et al., 2016	Yes	Yes	Yes	Yes	Yes	Australia	Research Paper
Walker et al., 2017	No	Yes	Yes	Yes	No	United States	Research Paper
Chorley, 2017	No	Yes	No	Yes	Yes	United Kingdom	Research Paper
Hortman-Hawthorne & Richards, 2017	No	Yes	Yes	Yes	Yes	United States	Literature Review
Shahmoradi et al., 2017	No	Yes	No	Yes	No	Iran	Research Paper
Parks et al., 2017	No	No	No	Yes	No	United States	Research Paper
Furusa & Coleman, 2018	No	Yes	Yes	Yes	No	Zimbabwe	Research Paper
Hong et al., 2018	Yes	Yes	No	Yes	Yes	No	Literature Review
Dong et al., 2018	No	Yes	No	Yes	No	United States	Viewpoint (Historical)
Alaqra et al., 2018	Yes	No	Yes	Yes	Yes	Germany; Sweden	Research Paper
Hylock & Zeng, 2019	Yes	Yes	No	Yes	Yes	United States	Research Paper
Dinh-Le et al., 2019	Yes	Yes	Yes	Yes	Yes	United States	Literature Review
Katusiime & Pinkwart, 2019	Yes	No	No	Yes	No	Uganda; Other developing countries	Literature Review
Zhang et al., 2019	Yes	Yes	No	Yes	No	No	Research Paper
Klecun et al., 2019	No	Yes	No	Yes	Yes	Singapore, England	Research Paper
Duan et al., 2020	Yes	Yes	No	Yes	No	United States	Research Paper
Tardif, 2020	No	No	No	Yes	Yes	Canada	Editorial

### Theme I: Anticipated Benefits

One of the most consistent themes across the literature is the potential of EHR to revolutionize multiple aspects of healthcare. This can be seen in the majority of articles in this review. In many of the articles published between 2005-2015, the role of information in 21st century healthcare is the main focus. Many of these papers describe how an EHR system can improve how patients are diagnosed and treated, and improve healthcare delivery in emergency rooms [2]. More recent literature (2015-2020) expands on this, detailing EHRs’ potential to contribute to medical research for populations, including for historical purposes. Both early and more recent papers also contrast the amazing potential of EHR with the high consequences of information leaks and abuse. Despite the uncertainty about privacy, there is a sense of optimism especially in the early literature about the capability of technology to eventually resolve the problem. Because of this, many early articles present a visionary view of how EHR could improve future medicine, alongside their discussion of contemporary risks and problems.

McClanahan (2008) describes how quick access to medical records through a universal EHR system could save thousands of patient lives each year in emergency rooms, due to a reduction of medical errors [3]. Other authors similarly emphasize the importance of information in medical decision making, and how EHR systems could vastly improve information delivery [2]. However they also describe how difficult it would be to create a “one-size-fits all” model of EHR on a national scale, and how some private companies offer services directly to patients and doctors in the absence of such a system [2].

Dinh-Le et al. (2019) note how EHR in the United States are overwhelmingly provided by private vendors, such as Epic, Cerner, and Meditech selling directly to doctors and patients. This paper focuses on wearable EHRs, and states that “a secure network, separate from the main hospital network, would need to be established to protect the privacy of wearable EHR” [4]. A task force paper by Jones et al. (2010) similarly notes how private vendors like Google and Microsoft sell personal health record (PHR) options to patients directly, which come in various formats, and have inconsistent levels of security. Ultimately such a system of isolated EHRs would lack the much needed interoperability and consistent privacy framework that a society reliant on EHR would require [5].

### Theme II: Patient-Doctor Trust

Patient and doctor communications in the context of EHR is another distinct theme that emerges in the literature. Baskaran et al. (2013) explore staff concerns about EHR, privacy, and patient consent at a maternity hospital in the United Kingdom. An EHR system for patient records could improve health outcomes and provide a means to assess healthcare quality for mothers on a national scale. However, the staff surveyed by Baskaran et al. express concern over the fact that sensitive health information could be illegally accessed by hackers [6]. This would potentially result in a loss of trust between the hospital and the public, and between patients and doctors.

Campos-Castillo & Anthony (2014), Patel et al. (2015), and Walker et al. (2017) all focus on how patients perceive the privacy of EHR, and how this influences their communication with their doctors [7-9]. They explore the issue of patients withholding medical information from doctors, due to their fears of data leaks from their records. All three of the above studies found that patients with a better understanding of EHR technology were less likely to withhold information, and that doctors could reduce information withholding by candidly discussing privacy issues and safeguards with patients, explaining the healthcare benefits of EHR, and by nurturing patient-doctor trust more generally. Vodicka et al. (2013) discuss how EHR could give patients access to their doctors’ notes, and allow them to be more involved in their own care. They state that this new potential for transparency “outweighs many patients’ privacy concerns” [10].

Concerns about EHR privacy are not only held by patients, but also by healthcare providers. Risks to patient-doctor confidentiality is cited as a drawback by Furusa & Coleman (2018), as well as by Shahmoradi et al. (2017). Doctors and other hospital staff interviewed in both studies expressed concerns over patient privacy risks, as well as the possibility of EHR disrupting normal workflow [11,12]. However, the hospitals featured in these studies also have smaller technical infrastructures, which complicates the mass-adoption of EHR. A similarity among Furusa & Coleman’s article and those by Campos-Castillo & Anthony, Patel et al., and Walker et al., is that they all suggest greater technological literacy can improve trust in an EHR system, as it leads to greater understanding of privacy measures, and greater patient and doctor control over content.

### Theme III: Tech/Privacy Conundrum

A third theme that appears is technology advancements to improve EHR privacy. This is especially prominent in the more recent literature (2015-2020). A variety of information technologies and strategies are presented. Vimalachandran et al. (2016) propose a hierarchical role-based authentication for EHR, which allows different levels of access to different roles within hospitals. This would reduce the risk of data leaks by restricting those who are not the patient or a medical professional from accessing the record [13]. Alaqra et al. (2018) also explore ways to enhance privacy and promote patient and doctor comfort with EHR. This study discusses cryptographic technology that allows for selective redaction of patient data stored on cloud servers [14]. Both Vimalachandran et al. and Alaqra et al. state that privacy of EHR increases when patients have greater control over their health records.

A study similar to Alaqra et al.’s (2018) paper is by Rodrigues et al. (2013). Both studies examine ways to improve security for EHR stored on the cloud. Public and private key encryption, as well as role-based authentication are presented as possible solutions [14, 15]. Cloud servers are presented in these papers as a way to store vast amounts of data, which would be essential for creating any large-scale integrated EHR system.

Hylock & Zeng (2019) write about ways to enhance the privacy of EHR through public and private key encryption. They propose blockchain as a way to store vast amounts of data while encrypting it. This could prevent information leaks resulting from unauthorized access. This study notes the potential of blockchain technology to improve privacy through detailed, public tracking of access. This could theoretically advance privacy beyond even paper records [16]. However, there are also innate privacy risks to digital records, arising from the ability to access them remotely, unrestricted by location. These inherent privacy risks are also explored by Katusiime & Pinkwart (2019). This paper reviews how patient-access of their EHR through mobile devices and tablets can expose personal health data to their network providers, hackers, and anyone who gains access to their device [17].

Hong et al. (2018) review the issue of big data in healthcare around the world, as well as the privacy issues arising from big data EHR systems. They discuss a variety of challenges, including the difficulty of sharing medical information between hospitals in China, controlling EHR access in the cloud, ethical questions of using EHR for population health research, and practical questions of storing unprecedented volumes of data [18]. They note that a big data system is necessary to accommodate vast numbers of patient records, but at the same time poses inherent privacy risks, for which there are no perfect solutions [18].

Duan et al. (2020) develop a mathematical algorithm to transfer health data from EHR while keeping patient information private. They demonstrate how this algorithm could allow for large-scale statistical analysis of EHR data for health research [19]. Another study by Zhang et al. (2019) explores a machine-learning approach to protecting EHR privacy. They look at generative adversarial networks (GANs) as a way to anonymize the patient details in EHR, and generate false records. These could be used to test a system’s safeguards against hacker attacks. Although this offers new possibilities for protecting privacy, the authors note that GANs are not perfect, and there is still a risk that genuine patient information could be identified [20].

### Theme IV: Ethics of Ownership

A final theme emerges in the LIS literature in the question of EHR ownership. The utility of EHR over the years have expanded beyond the delivery of healthcare itself, to include the ability to analyze population health through data analysis and informatics. Hong et al. (2018) explore how EHR can be used to study population health and epidemiology [18]. Another study by Dong et al. (2018) shows how historical medical records can be analyzed to advance sociological and medical history research [21]. However, both papers acknowledge the ethics and privacy issues linked to both of these secondary uses.

The article by Dong et al. does not focus on the ownership question explicitly, because it is an archival paper, which focuses on historical patient records and the potential benefits of digitizing them [21]. However, the ownership question is inherently linked to the ethics of secondary uses, especially for population health and historical medicine research. Could, for example, a family member of a patient in one of the records request the removal of the individual’s digitized health record from the internet? Could they request its removal from the research pool completely? If so, what would be the ethical implications for health and historical research using EHR or digitized patient records? These questions are speculative, however they elaborate some possible ownership challenges of EHR and privacy.

A paper by Chorley (2017) illustrates the ethical conundrum when a government owns hospital data, including EHR, and has the right to publish such data on the internet through Freedom of Information laws [22]. The great benefits to public health research lie in contrast with patients’ rights to privacy. A deep lack of trust in the EHR system develops among the people it is intended to help. Additionally, Hortman Hawthorne & Richards (2017) discuss the uncertainties over EHR ownership and stewardship [23]. They describe how personal health records (PHR), owned and controlled by patients, may be the legal and ethical answer to this uncertainty.

Uncertainties around privacy obligations complicate the question of ownership. Tardif (2020) discusses the complex responsibilities of “healthcare information custodians” [24]. He describes how healthcare providers in Canada can misunderstand privacy laws around EHR, and how this can violate patients’ consent of who views their data [24]. Klecun et al. (2019) examine EHR implementation from an institutional perspective. They mention the inherent difficulty of informing all stakeholders of their privacy obligations across a nation-wide system [25]. Another study by Parks et al. (2017), argues that disruptions in workflow happen when a hospital has to manage the needs of so many different patients with individual access restrictions to their records [26]. The questions of privacy and ownership of EHR are mentioned consistently over the past fifteen years of literature, and across various types of papers and fields within LIS. However due to the complex ethical nature of these questions, and the divergence of opinions, these issues remain unresolved in the present day.

## Discussion

A unifying opinion among the reviewed papers is the potential for the improvement of healthcare by the further development and implementation of EHR. However, EHR is fraught with both technical and privacy ethics limitations, which complicate its realization across different countries and healthcare systems. EHR requires a relatively strong and robust IT infrastructure in the hospital it is being implemented in; something not always available in developing economies [11]. Additionally, even when the technical capacity exists, there is resistance among healthcare professionals, staff, and patients over privacy risks [14]. The full potential of EHR is additionally held back by improper use by health professionals, companies, and governments, which results in lack of trust and resistance to their use [14].

EHR has an inconsistent implementation across countries, partially due to variations in cultural attitudes on information collection and privacy. This extends to societies with the same laws governing information and privacy protections such as countries in the European Union. In Sweden there are higher levels of trust and implementation of EHR. However, in Germany there are lower levels of both patient trust in EHR and medical professionals’ confidence in them [14]. This highlights relevant questions of trust between patients, healthcare professionals and the amount of control governments can and should have over patient information. Future studies should continue to explore the nature of the relationship between healthcare professionals and patients, and their governments.

Another challenge that arises in the research is the resistance to EHR by both patients and medical professionals due to privacy concerns. The concern over privacy is a consistent and unifying feature in all papers in this review. There are significant ethical questions as to how much, and whether, patient privacy is worth risking. There appears to be a general consensus that accurate, accessible and detailed records have an inverse relationship with privacy. Although technology has advanced enormously over the past fifteen years, leading to improved data capacity and security measures, nearly all authors of the included papers acknowledge that the privacy risks of EHR is never zero. As a result, privacy remains the most significant barrier to a universal EHR system. It will be interesting to observe how privacy technology continues to develop over the coming years, and if privacy can be sufficiently protected to allow for such a system.

The question of EHR and ownership is highly complex, and has significant implications for privacy and ethics. We believe this is an area of the literature that merits further research. Firstly, the ownership of individual EHR brings up questions of content control and accuracy. One of the clear benefits of EHR is the facility of transferring information. However, maximum benefit from this requires the health information itself to be accurate and complete. In the literature we reviewed, there was limited discussion of the problem of human error, and how potential misinformation, including misdiagnoses and doctor bias, could be transmitted through EHR. This is one potential problem that can arise through hospital or clinic ownership of EHR. Further, patient ownership could create other problems, such as critical information being altered or removed due to patients misunderstanding of specialized medical terminology [10, 23].

Another challenge of ownership is the fact that many modern-day EHR are dependent on privately owned software and hardware to store, format and encrypt them. Problems arise when the technology necessary for any of these functions becomes obsolete or otherwise inoperable. For example, EHR being formatted and encrypted using a proprietary file format are vulnerable to its creators being unable or unwilling to provide updates to keep the files up-to-date. The company could move away from the service or go out of business, leaving institutions vulnerable to data loss. Furthermore, flaws in the software or hardware can lead to a loss in the integrity of the data, or exposed security risks leading to the loss of records. The question of ownership and content control should be further explored, as this has major implications for privacy.

Finally, the ethics of secondary uses of EHR data deserves greater attention. EHR has excellent possible applications in medical history and epidemiological research. However, given that laws vary between countries and in their protections for patients, there exists a temptation to use personal health data for commercial purposes. An example of this is a 2019 story by the Toronto-based newspaper, The Toronto Star. The article describes the sale of patient medical records to an American health data company. This resulted in an investigation by The Office of Information and Privacy Commissioner of Ontario [27]. The secondary use of patient data also brings up the question of what rights if any do patients have over the use of their EHR? The ethical dilemma of privacy vs the public good is highlighted by EHR and their secondary uses. As reviewers, we are eager to see how the conversation on this issue evolves in the coming years.

### Limitations

There are limitations to this review. This review relied entirely on the two databases LISA and LISTA, and used a limited set of search terms that may have unintentionally left out relevant and meaningful research that has been conducted in this field. Additionally, all articles selected were written in English, thus excluding a variety of insightful works in other languages. In addition, despite the international focus of the review, there is no direct representation of EHR in Latin America.

### Conclusion

EHR is and will continue to be used in healthcare, and has the ability to advance the health field in a multitude of positive ways. However, its status, use and limitations, especially regarding privacy, are far from certain. Healthcare and information professionals are grappling with complex questions regarding the benefits and risks of EHR. It is clear that for EHR to be most effective, it comes at the cost of patient privacy rights and exposes patients to the consequences of security breaches. In this paper, we identified how EHR and privacy link to complex issues of communication, technology, ownership, and the future of healthcare information. We hope that this literature review will provide insight into how EHR and privacy have been studied in the LIS field, and impart an appreciation for the complexity of these issues. Although these themes are multi-layered and ethically deep, they are fundamentally of interest not only to health and information professionals, but also to interested patients, the general public, and for policy makers grappling with this issue.

## Footnotes

1 ***Terminology Note:*** Some papers use the terms electronic medical records, electronic health records, and electronic patient records interchangeably, while others draw distinctions between their meanings. For the purpose of consistency and clarification, we use the term electronic health records, shortened to “EHR”, in this paper to refer to both individual records and integrated records systems. EHR is the most commonly used term across the literature we selected.
